# Investigation of Dye Dopant Influence on Electrooptical and Morphology Properties of Polymeric Acceptor Matrix Dedicated for Ternary Organic Solar Cells

**DOI:** 10.3390/polym13234099

**Published:** 2021-11-25

**Authors:** Gabriela Lewińska, Piotr Jeleń, Jarosław Kanak, Łukasz Walczak, Robert Socha, Maciej Sitarz, Jerzy Sanetra, Konstanty Waldemar Marszałek

**Affiliations:** 1 Faculty of Computer Science, Electronics and Telecommunication, AGH University of Science and Technology, 30 Mickiewicza Ave., 30059 Krakow, Poland; kanak@agh.edu.pl (J.K.); marszale@agh.edu.pl (K.W.M.); 2 Department of Silicate Chemistry and Macromolecular Compounds, Faculty of Materials Science and Ceramics, AGH University of Science and Technology, 30059 Krakow, Poland; pjelen@agh.edu.pl (P.J.); msitarz@agh.edu.pl (M.S.); 3 Science & Research Division, PREVAC sp. z o.o., Raciborska 61, 44362 Rogow, Poland; lukasz.walczak@prevac.pl; 4 Jerzy Haber Institute of Catalysis and Surface Chemistry, Polish Academy of Sciences, Niezapominajek 8, 30239 Krakow, Poland; robert.socha@ikifp.edu.pl

**Keywords:** dye, organic solar cells, ellipsometry, ternary organic films, morphology examination

## Abstract

The publication presents the results of investigations of the influence of dye dopant on the electrooptical and morphology properties of a polymeric donor:acceptor mixture. Ternary thin films (polymer:dye:fullerene) were investigated for potential application as an active layer in organic solar cells. The aim of the research is to determine the effect of selected dye materials (dye D131, dye D149, dye D205, dye D358) on the three-component layer and their potential usefulness as an additional donor in ternary cells, based on P3HT donor and PC71BM acceptor. UV–vis spectroscopy studies were performed, and absorption and luminescence spectra were determined. Ellipsometry parameters for single dye and ternary layers have been measured. The analyses were performed using the Raman spectroscopy method, and the Raman spectra of the mixtures and single components have been determined. Organic layers were prepared and studied using scanning electron microscope and atomic force microscope. For dyes, ultraviolet photoelectron spectroscopy and X-ray photoelectron spectroscopy studies were carried out and the ternary system was presented and analyzed in terms of energy bands.

## 1. Introduction

Organic photovoltaics (OPV) has been developing in recent years due to its cost, solution processibility, semi-transparency, mechanical flexibility, light weight, and has become increasingly interesting for technology [1,2,3]. The possibility of application in wearable systems, connection to IOT (Internet of Things) and powering personal devices [4,5,6] as well as the hope for the use of organic cells in space [7], causes substantial popularity of the organic photovoltaic. It should also be mentioned the possibility of using OPV in indoor applications, which have excellent potential for use in flexible and wearable electronics [8,9,10]. One of the rising trends in organic photovoltaics are ternary solar cells.

In ternary solar cells [11], a suitably selected third component (additional donor or additional acceptor) is introduced into the active layer to increase cell efficiency. The mixture (donor 1:donor 2:acceptor blend) forms a bulk heterostructure and in the photovoltaic process, and photons are absorbed. Materials that broaden the absorption spectrum and facilitate the transport of holes and electrons are selected as the third element [12]. A beneficial effect of the extra component on the morphology of the thin film has also been observed.

From the first mention of two-component active layer additives and three-component systems appeared around the mid-2000s. Since then, quite a few ternary systems have been synthesized and tested, with efficiency increasing from 2% to about 17% [13,14].

In ternary systems, different chemical compounds were used because of their molecular structure (polymers or small molecules). The output efficiency of the binary system was increased by 12% (in the ratio of P3HT: PCBM PCPDTBT 0.8:1:0.2) using the addition of the polymer poly[2,6-(4,4-bis-(2-ethylhexyl)-4H-cyclopenta[2,1-b;3,4-b′]-dithiophene)-alt-4,7-(2,1,3-benzothiadiazoles)] (PCPDTBT) [15]. In 2015, a efficiency over 5% (a 20% improvement, relative to the binary system) was achieved on the doping of naphthalene azomethylenediimides [16]. Another cells operating using poly[4-(5-(4,8-bis(dodecyloxy)-4,8-dihydrobenzo[1,2-b:4,5-b′]dithiophen-2-yl)-alt-5,8-bis-(thiophen-2-yl)-6,7-bis(3,4-bis(dodecyloxy)phenyl)-2-dodecyl-2H-[1,2,3]triazolo[4,5-g]quinoxaline] (PBDT- BTzQx-C12) with BDT as the donor building block and TzQ_x_ as the acceptor building block. These systems (depending on the percentage amount from 1 to 4%) achieved efficiency from 2.73 to 3.54% [17]. Poly[[4,8-bis[(2-ethylhexyl)oxy]benzo[1,2-b:4,5-b′]dithiophen-2,6-diyl][3-fluoro-2-[(2-ethylhexyl)carbonyl]thieno[3,4-b]thiophenediyl]] (PTB7)-based cells present some of the highest reported efficiencies for polymer fullerene solar cells due to enhanced near-infrared absorption and lower HOMO. Polymer-doped cells in the donor-acceptor configuration of PTB7: PCBM showed efficiencies above 8.6% [18] and 10.4 % [19].

Dyes are also a type of additive being developed and studied in ternary systems. Their main advantages are (usually) high values of absorption coefficients. Many dyes are characterized by absorption spectra in the sunlight range. For P3HT:PCBM systems doped with dyes bis(trihexylsilyl oxide) silicon phthalocyine (SiPc) and bis(trihexylsilyl oxide) silicon naphthalocyanine (SiNc). The addition of both compounds increased the energy conversion efficiency to 4.3% compared to single dye ternary solar cells when illuminated with the AM1.5G spectrum SiPC:P3HT:PCBM and SiNC:P3HT:PCBM systems achieved 4.1% and 3.7%, respectively [20]. The SiPC derivative silicon bis(6-azidohexanoate)phthalocyanine ((HxN3)2-SiPC) with crosslinking groups was also used to obtain cells with an efficiency of 3.4% [13]. High efficiencies were achieved by ternary systems with the addition of small-molecule hinges efficiencies of 7–10% [21,22,23].

This paper presents a study of ternary mixtures of polymer:dye:fullerene for potential applications as an active layer in ternary organic cells [24,25]. The introduction of an additional substance (donor-dye) is aimed at reducing the roughness and improving the quality of the layer. The aim of the study was to check the influence of the investigated compounds on the morphology of layers in ternary systems.

The dye materials selected were chosen for their requested properties. Surface sensitization dyes are standard process for DSSCs, the aim of this research is to investigate them in the context of applications in organic electronics, more specifically in organic photovoltaic cells. It is also required to consider their potential for use as colors controller in a polymer matrix [26], in organic transistors [27] or as organic memories [28].

The typical active layer in organic cells is a mixture of donor and acceptor materials (bulk heterojunction, BHJ). BHJ cells are much cheaper and simpler to construct, in comparison to bilayer devices as they can be applied from wet phase. In the case of ternary solar cells, we are dealing with three chemical compounds. It is therefore important to select a common solvent. In an ideal case, the acceptor is homogeneously dispersed in the matrix formed by the donors, thus forming a three-dimensional network. The mixture allows a large increase in the contact area between these materials, leading to an increase in the number of excitons produced, as well as facilitating their dissociation into free charge carriers.

## 2. Materials and Methods

**Figure 1:**
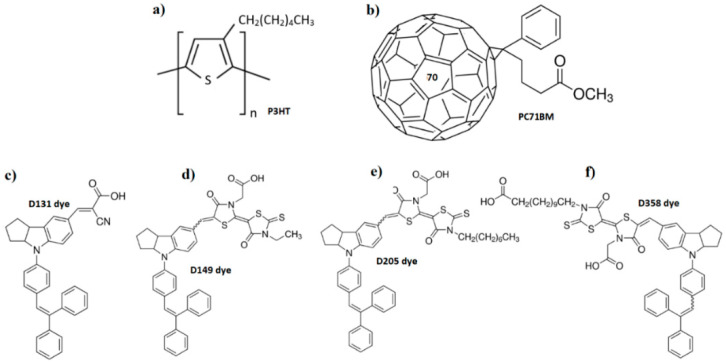
Chemical formula of (**a**) poly(3-hexylthiophene-2,5-diyl) (P3HT), (**b**) phenyl-C71-butyric acid methyl ester (71) and dye materials under investigation: (**c**) dye D131, (**d**) dye D149, (**e**) dye D205, (**f**) dye D358.

Thiophene-based compounds, polyacetylene derivatives, or polyanilines are used as donor materials, among others. Compounds based on fullerene or perylene diamides are commonly used as acceptor materials. In this case, poly(3-hexylthiophene-2,5-diyl) (P3HT) was used as a donor material, phenyl-C71-butyric acid methyl ester (PC71BM) [29] was chosen as an acceptor (the chemical formulas are shown in Figure 1a,b). Series of four dye materials were tested as dopants (potential second donors) in the study: dye D131 (2-cyano-3-[4-[4-(2,2-diphenylethenyl)phenyl]-1,2,3,3a,4,8b-hexahydrocyclopent[b]indol-7-yl]-2-propenoic acid), dye D149 (5-[[4-[4-(2,2-diphenylethenyl)phenyl]-1,2,3-3a,4,8b-hexahydrocyclopent[b]indol-7-yl]methylene]-2-(3-ethyl-4-oxo-2-thioxo-5-thiazolidinylidene)-4-oxo-3-thiazolidineacetic acid), dye D205 (5-[[4-[4-(2,2-diphenylethenyl)phenyl]-1,2,3,3a,4,8b-hexahydrocyclopent[b]indol-7-yl]methylene]-2-(3-octyl-4-oxo-2-thioxo-5-thiazolidinylidene)-4-oxo-3-thiazolidineacetic acid) and D358 (5-[3-(carboxymethyl)-5-[[4-[4-(2,2-diphenylethenyl)phenyl]-1,2,3,3a,4,8b-hexahydrocyclopent[b]indol-7-yl]methylene]-4-oxo-2-thiazolidinylidene]-4-oxo-2-thioxo-3-thiazolidinedodecanoic acid). They were provided by Merck KGaA (Darmstadt, Germany), along with other reagents. The information about dyes were collected in Table S1 in Supplementary Materials. Chemical formulas of the tested dye materials are shown in Figure 1c–f [30]. The materials were provided in solid form (colored powder). The solutions were made at concentrations of 10 mg/mL. In this study, the materials were dissolved in spectroscopically grade chloroform.

Spectroscopic studies were performed using Avantes Sensline Ava-Spec ULS-RS-TEC fiber optic spectrophotometer (Avantes, Appelsdorn, The Netherlands) with Avantes AvaLight DH-S-BAL-Hal lamp. Absorption and luminescence spectra of solid were investigated as a thin film (quartz was used as a reference) within the range 250–1100 nm. Photoluminescence tests were carried out using laser excitation at wavelengths $\lambda_{ex}=405\;\mathrm{nm}$ and $\lambda_{ex}=532\;\mathrm{nm}$. Raman measurements were performed using Witec Alpha 300 M+ spectrometer (WITec, Ulm, Germany) equipped with the 488 nm laser, 600 groove grating, and a 100× ZEISS objective (Oberkochen, Germany). Laser power was adjusted to prevent sample degradation. The samples were deposited on a glass substrate.

For spectroscopic ellipsometry, analysis was carried out using Woolam M-2000 ellipsometer (Lincoln, NE, USA), with a wavelength range from UV to near-infrared. Atomic force microscope pictures were taken using the NTMDT Ntegra Aura (Apeldoorn, The Netherlands) system in SemiContact mode. Scanning electron microscope (SEM) analyses were done using ultra-high resolution scanning electron microscopy with field emission (FEG—Schottky emitter) NOVA NANO SEM 200 (Hitachi, Japan) cooperating with an EDAX EDS analyzer.

The X-ray photoelectron spectroscopy/ultraviolet photoelectron spectroscopy (XPS/UPS) experiment (PREVAC sp. z o.o., Rogow, Poland) was performed using the XPS/UPS/ARPES PREVAC setup, in an ultra-high vacuum (UHV) chamber with a base pressure around 8 × 10^−10^ mbar and at room temperature. The analysis chamber was equipped with a Ea15 PREVAC hemispherical analyzer and UVS 40B source PREVAC (UV power U = 0.56 kV, PUV = 55 W, He I). Binding energy (BE) scale was calibrated at the Fermi level 16.87 eV.

## 3. Results and Discussions

### 3.1. Optical Properties

**Figure 2:**
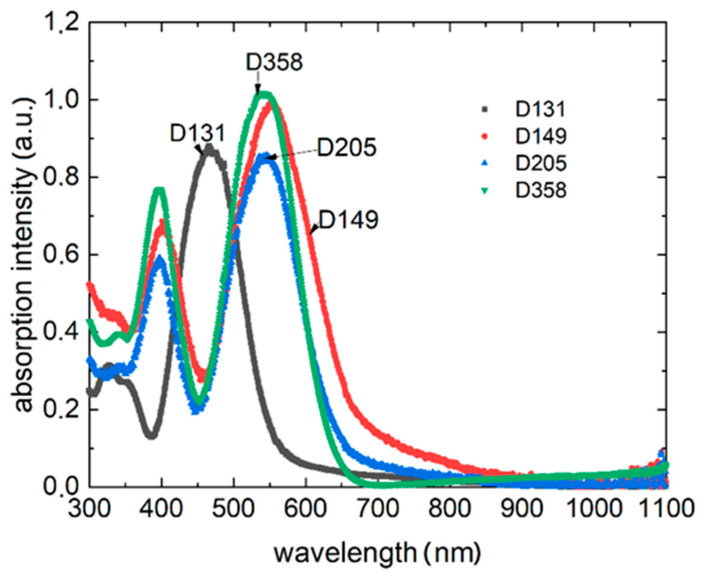
Absorption spectra of the investigated dyes.

The absorption spectra for all compounds considered are presented in Figure 2. Each of the compounds showed a broad absorption spectrum, in the case of D131 with one maximum, the others show the presence of two maxima. D139 has a distinct absorption maximum at 464 nm. The other three compounds have dual-maximum spectra, located for dye D149:400 nm, 560 nm, dye D205:395 nm, 545 nm, dye D358:398 nm, 545 nm, respectively. Examining the absorption edges of the compounds, the energy gaps were determined as follows: for D131 Eg = 2.2 eV, for D149 Eg = 1.8 eV, for D205 Eg = 1.9eV, and for D385 Eg = 1.9 eV.

**Figure 3:**
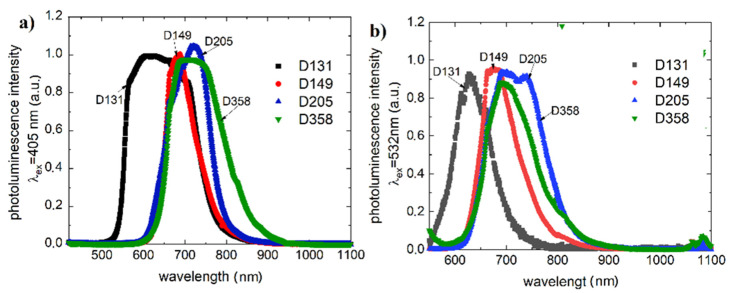
Photoluminescence spectra of the studied dyes (**a**) $\lambda_{ex}=405\;\mathrm{nm}$, (**b**) $\lambda_{ex}=532\;\mathrm{nm}$.

All dyes exhibit clear photoluminescence (Figure 3) for blue and green laser excitation wavelength excitation $(\lambda_{ex}=405\;\mathrm{nm}$ and $\lambda_{ex}=532\;\mathrm{nm}$).

For compound D131 in the case of excitation (405 nm) we obtained a maximum indicating (photoluminescence) state at 559, 607, 664 and 708 nm. The green laser-induced (532 nm) spectrum does not show the first maximum. The other excitations taper slightly at 611, 623, and 708 nm, respectively. For the D149 dye, the absorption maxima shown for both excitations are: 661, 673, and 688 nm. The luminescence spectrum for dye D205 excited by blue laser shows two minor maxima at 662 and 685 nm and expressed at 720 nm. Treated with green excitation, the spectrum of D205 splits into two distinct luminescence bands with maxima at 694 and 739 nm. The maximum observed for 405 nm excitation is almost invisible. The luminescence spectrum of D358 has small maxima around 660 nm and main band with a maximum of 689 nm. It is observed for both investigated excitations, however, in the case of green laser excitation the luminescence band narrows.

**Figure 4:**
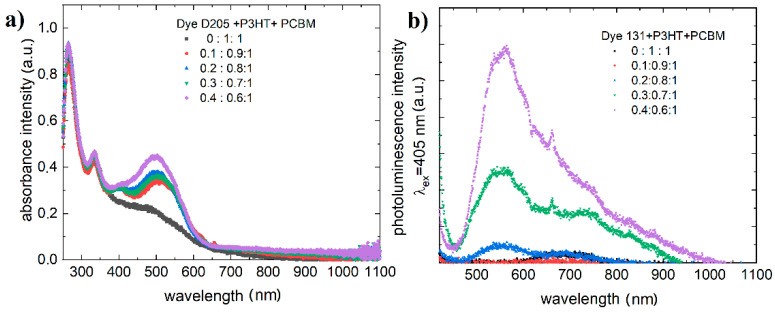
Absorption (**a**) and photoluminescence (**b**) ($\lambda_{ex}=405\;\mathrm{nm}$) spectra for mixtures dye:P3HT:PCBM.

Figure 4 shows the absorption and photoluminescence $(\lambda_{ex}=405\;\mathrm{nm}$) spectra for example dyes for different weight ratios of dye to donor (P3HT). The weight ratio of donors to acceptor (PCBM) was 1:1.

The photoluminescence coming from the P3HT:PCBM mixture is quenched by absorption of D131 (Figure 4) and only emission from D131 is visible (Figure 3).

**Figure 5:**
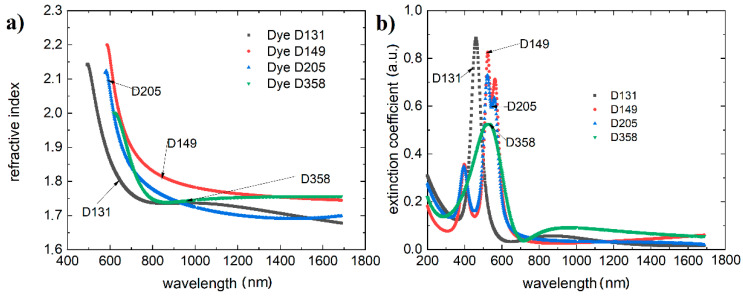
(**a**) Refractive indices versus wavelength; (**b**) extinction coefficients versus wavelength for the investigated materials.

The results of the dispersion relations for the refractive indices (n) of the studied compounds and for the extraction coefficients (k) are presented in Figure 5. The mode used to model the active layer was general oscillators (Gen-Osc) composed of Cody–Lorentz and Tauc–Lorentz type oscillators after initial spline modeling.

The Lorentz-type oscillator and derived oscillator model were used during fitting. The classical harmonic oscillator model is very similar to the Lorentz oscillator, but is derived from the quantum theory of mechanical perturbations.

(1) $$\varepsilon \left(E\right)=\varepsilon_{1}\left(E\right)-{i\varepsilon }_{2}\left(E\right)=\varepsilon_{\infty }+\frac{2{\mathrm{AE}}_{0}}{E_{0}^{2}-E^{2}+i\Gamma E+\frac{1}{4}\Gamma^{2}}$$

In Equation (1), A is approximately the peak of $\varepsilon_{2}$ at the resonant energy and Γ is the full width at half the maximum value found with the Lorentz oscillator. Tauc–Lorentz [31] (Equation (2)) and Cody–Lorentz [32] (Equation (3)), models provide a more realistic representation of real materials and are widely used to describe many amorphous dielectrics and semiconductors (a detailed description can be found in Supplementary Materials).

The Tauc–Lorentz absorption formula is as follows:

(2) $$\varepsilon_{2}\propto \frac{{\left(E-E_{g}\right)}^{2}}{E_{g}^{2}}$$

and the Cody–Lorentz model is shown by the equation:

(3) $$\varepsilon_{2}\propto \;{\left(E-E_{g}\right)}^{2}$$

All dyes exhibit complex multi-oscillator spectra.

The simplest single-maximal dispersion spectra of extinction coefficients have been recorded for dyes D131 and D358, which corresponds to the absorption spectra. The spectra of D149 and D205 show a multipeak character. With respect to the dispersion dependence of the refractive index, they show differentiated maxima for D131 at 496 nm reaching 2.14, for D149 at 587 nm reaching 2.19, D205 at 585 nm having a refractive index of 2.12 and compound D358 at 625 nm having a refractive index of 2.00. The dispersion dependence of the refractive indices after passing through the maximum is stabilized at the level of about 1.7–1.8. The refractive index and extinction coefficient values for the 633 nm wavelength are summarized in Table 1. The thicknesses of the layers tested in can be found in the Supplementary Materials (Table S2).

**Figure 6:**
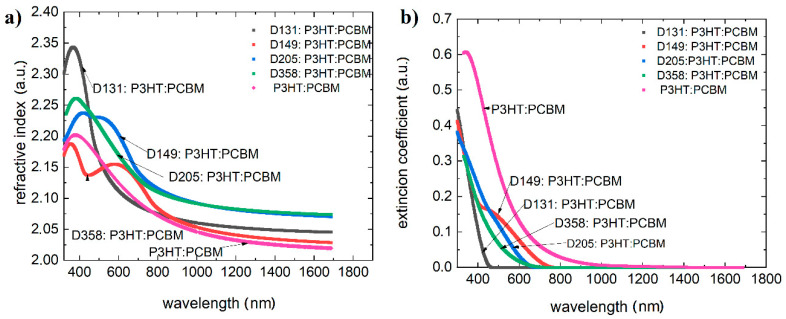
(**a**) Refractive indices versus wavelength, and (**b**) extinction coefficients versus wavelength for mixtures dye:P3HT:PCBM.

Due to the application potential, we found the effect of dye in ternary systems (with PCBM and P3HT) on the optical properties of the active layer (Figure 6).

### 3.2. Surface Morphology

**Figure 7:**
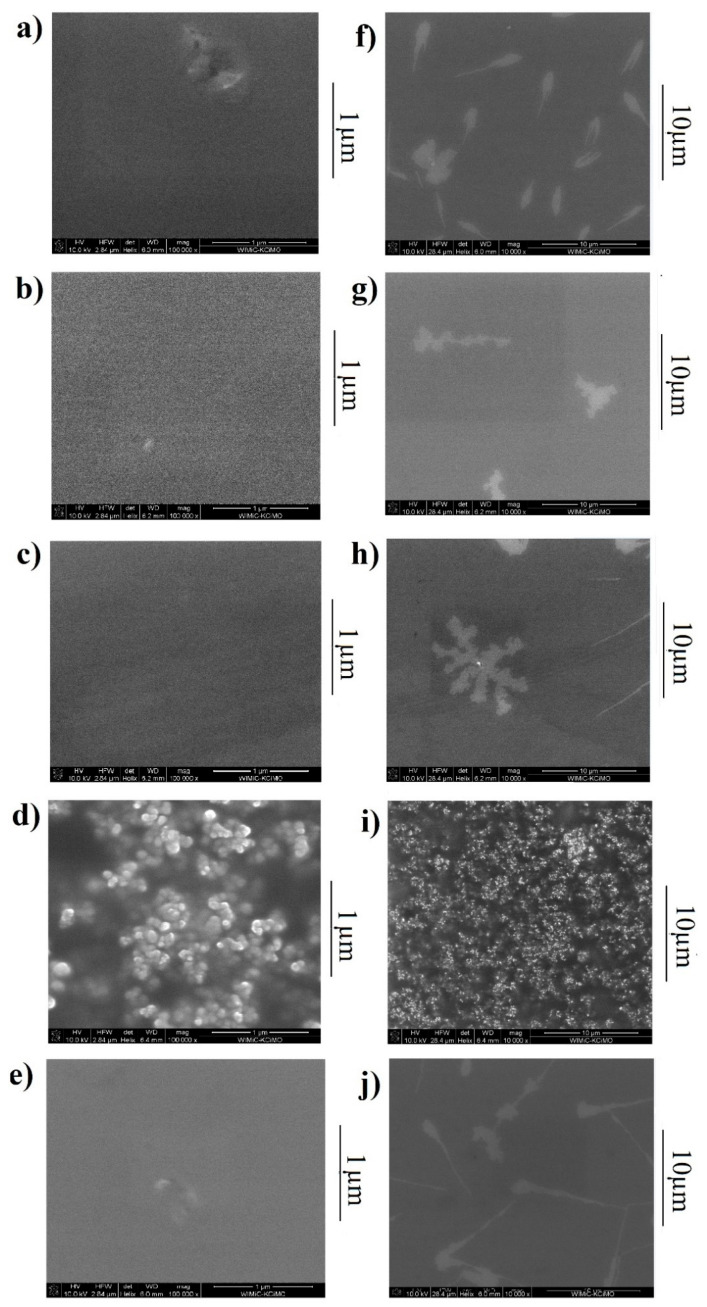
SEM images of the tested mixtures: (**a**,**f**) P3HT:PCBM; (**b**,**g**) dye D131:P3HT:PCBM; (**c**,**h**) dye D149:P3HT:PCBM; (**d**,**i**) dye D205:P3HT:PCBM; (**e**,**j**) dye D358:P3HT:PCBM. Left row shows images taken at a magnification of 100,000× times, the right row at a magnification of 10,000× times.

**Figure 8:**
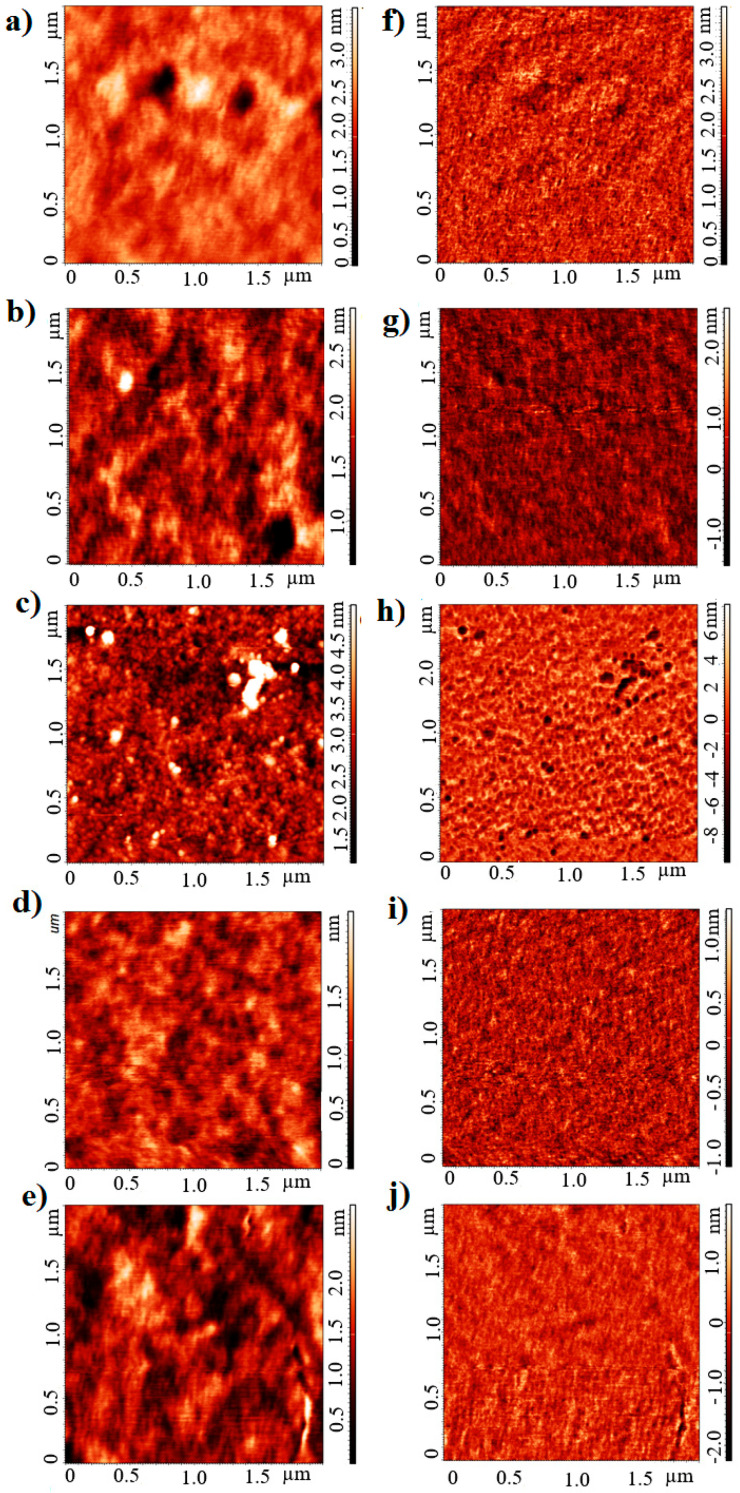
AFM images of the tested mixtures (**a**,**f**) P3HT:PCBM; (**b**,**g**) dye 201 D131:P3HT:PCBM; (**c**,**h**) dye D149:P3HT:PCBM; (**d**,**i**) dye D205:P3HT:PCBM; (**e**,**j**) dye 202 D358:P3HT:PCBM. Left row shows the morphology, the right row presents phase contrast.

To evaluate the surface morphology, atomic force microscopy (AFM) and scanning electron microscopy (SEM) were performed. The study of ternary systems was carried out with reference to the donor-acceptor system. Images of the layers under consideration are shown in the AFM: Figure 7 and SEM: Figure 8. AFM profiles are included in Figure S1 (Supplementary Materials).

The results at 10,000× magnification confirm that the obtained layers are smooth and well distributed (apart from negligible surface defects). At 100,000× magnification, the structure of the thin film is a mixture of elongated, slightly twisted elements (similar to fingerprint texture). The mixture in which clear structures are observed is a mixture with the dye D205:P3HT:PCBM. Strong inhomogeneities and emerging spherical formation can be seen. It can be concluded that in the case of D131, D149 and D358 substances we obtain a mixture in which the second donor is embedded in the donor, both donors are distributed in the acceptor [33,34,35].

The layer roughness’s obtained from the AFM measurements are summarized in Table 2. The addition of a third dye component (with the exception of D205), reduces the layer roughness.

### 3.3. Raman Spectroscopy

**Figure 9:**
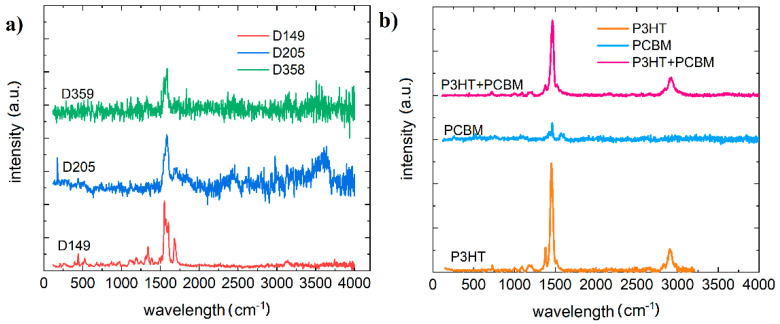
Raman plot (**a**) for dyes D149, D205, and D358 (**b**) P3HT, PCBM, and mixture P3HT and PCBM.

Due to the low robustness of material D131, it was not possible to obtain a Raman spectrum. Material D205 also showed slight degradation. The degradation of dye 131 is likely related to the fact that there is no sulfur in the compound, which affects the thermal properties, unlike the other investigated dyes [36]. The resulting spectra are shown in Figure 9.

**Figure 10:**
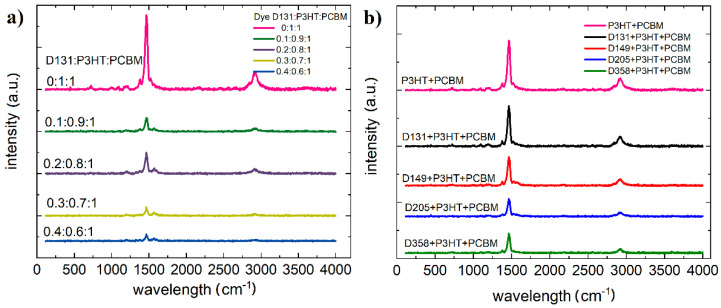
Raman plot (**a**) of dye D139:P3HT:PCBM ternary mixtures with different concentrations (**b**) of dye:P3HT:PCBM ternary mixtures for the investigated dyes. The intensity of the peaks has been normalized.

Pure PCBM and P3HT test was carried out by Yadov et al. [37,38]. For P3HT C–S–C ring deformation (725 cm^−1^), C–C intra-ring stretching 1379 cm^−1^ and symmetric C=C stretching vibrations (1447 cm^−1^) were identified. PCBM Raman spectrum shows four peaks located at 658, 1038, 1127 and 1570 cm^−1^. The intensity of the P3HT peaks is much higher than that of the PCBM, so that mainly the peaks of P3HT are visible in the mixture. Due to the extended range of measurements, a peak was also recorded at 2920 cm^–1^ corresponding to sp3 C−H stretching mode. The quantitative effects of the dye material and the effects of the investigated materials on the ternary system are shown in the Figure 10.

All dye compounds have intense vibrations of strong double bonds (C=C) around 1453 cm^−1^. Dye D149 shows peaks at 1590, 1619, and 1713 cm^−1^ and D205 at 1650 cm^−1^ and D358 at 1515 cm^−1^ and 1605 cm^−1^. This is identified with the vibrations of two carbon atoms linked by strong double bonds (C=C). In three-component mixtures this is quenched by the 1465 cm^−1^ vibration from P3HT.

### 3.4. X-ray Photoelectron Spectroscopy/Ultraviolet Photoelectron Spectroscopy

**Figure 11:**
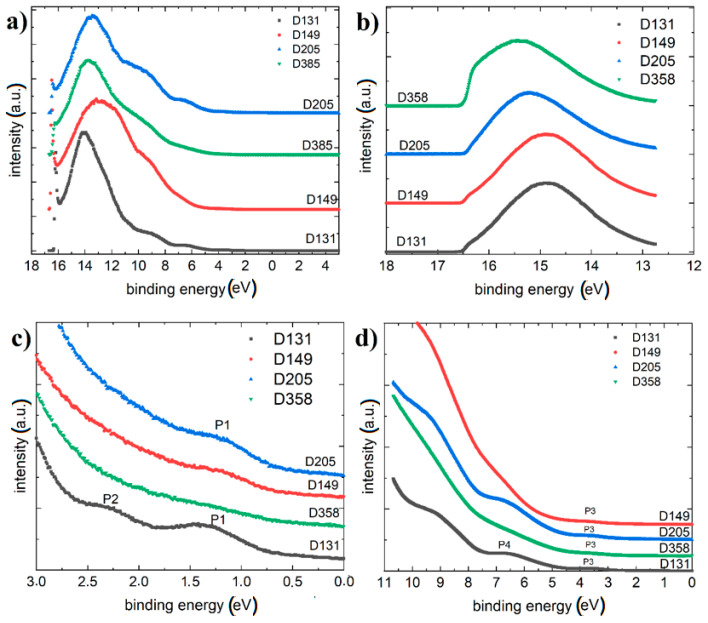
UPS results for investigated materials (**a**) survey spectrum for investigated materials (**b**) comparison of the cut-offs for all samples (**c**), UPS spectrum of the valence bands for all samples (**d**).

The He I UPS spectra of thin films investigated materials deposited on Au (1 1 1) were presented in Figure 11. We obtained a survey spectrum for investigated materials (shown in Figure 11a). A comparison of the cut-offs for all samples is also provided (Figure 11b). The obtained valence spectrum in different ranges is shown in Figure 11c,d. On the diagrams the postulated subsequent quantum states (P1–P4) are marked.

The S 2p spectra (Figure 11a) show main electronic state of sulfur at electron binding energy of approximately 164.5 eV, which is assigned to sulfur in thiazolidine ring. The lower maximum at 162.3 eV is ascribed to thioketone group (S=C). Additionally, some oxidation of sulfur to sulfate species is indicated by slight increase of background above 168 eV. The latter is observed well for D358 compound but it is insignificant for the other ones.

**Figure 12:**
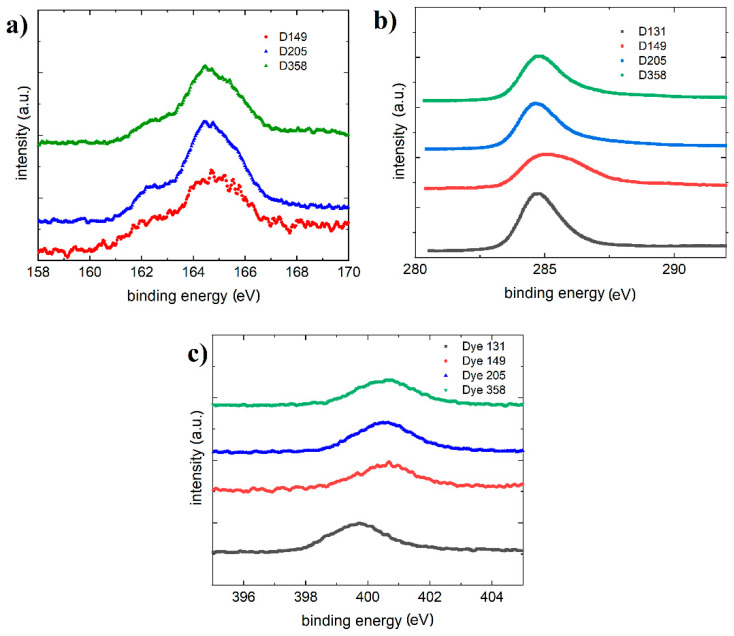
(**a**) The S 2p core excitations of D149, D205 and D358 thin films surface (**b**) the C 1s core excitations (**c**) the N 1s core excitations of D131, D149, D205 and D358 thin films surfaces.

The C 1s (Figure 12b) spectra are very similar for D131, D205 and D358 compounds but different for D149. The difference is related to a shoulder at approximately 287 eV, which indicates presence of carbonyl/ketone groups (C=O) well related to dye structure. On the other hand, D205 has similar structure but the C 1s spectrum differs significantly of D149. Such difference can be an effect of much longer aliphatic chain (8 carbons) of D205 than in case of D149 (1 carbon).

The N 1s (Figure 12c) spectra show similar shape of all spectrum envelopes with some negative shift of the intensity maximum for D131. The shift is app. 1 eV and it is correlated with lower electronegativity of nitrogen in the indole structure than in thiazolidine ring. Additionally, these spectra suggest that indole structure is rather screened by thiazolidine ring that can indicate external location of the latter one at the D149, D205 and D358 samples surfaces.

## 4. Discussion

Investigated dyes have broad absorption spectra, overlapping with the spectrum of sunlight, which makes it suitable for use as a donor element. Dyes were previously simulated using the Cauchy model [39] and EMA-layer [40], however, for the compounds under consideration, the oscillator model [41,42] was chosen because of the broad absorption band.

The law of additivity of absorbance [43] is used in the spectrophotometric analysis of multicomponent systems. If there are more substances in a solution that absorb radiation at a selected wavelength, then the absorbance of this solution is equal to the sum of the absorbances of its individual components. From this point of view, the inserting of additional components is beneficial to produce the most absorbent material.

It can be seen that the photoluminescence increases with the concentration of the dye. This type of effect is observed up to a certain concentration [44]. Absorption of the dye in the region of 400 to 600 nm further absorbs the emission of the P3HT:PCBM mixture [45].

According to the energy levels, the acceptor picks up all the excited electrons causing all the luminescence coming from dyes and P3HT to be quenched. Subsequent studies have indicated however, that this relationship deviates from the linear function, even for non-interacting compounds [46]. For multicomponent systems, an analysis that considers the Kramers–Kronig relation is warranted, linking concentration to oscillator strength. PCBM and P3HT mixtures are quite well established. Their spectra of extinction coefficient and refractive index were studied by Ng et al. [47]. Furthermore, the addition of a third component shows that the reflection coefficient of the active layer is also reduced. The antireflective property is also very favorable.

Surface tests on P3HT: PCBM have already been carried out by Hajduk et al. [48]. Increasing the donor proportion, caused an increase in roughness. AFM images showed a smoother surface for binary mixtures with a lower amount of donor (P3HT) [49]. We therefore focused on observing the effect of third component on the donor:acceptor system. Improved quality (reduced roughness) is the expected result [50]. Only the additive dye D205 is not promising, due to the structure formation. The other layers present a blended structure. The homogeneity of the layer has an additional advantage: exciton in the mixture is generally short-lived, so the size of the individual domains is therefore critical. It is also essential that the domains are interconnected so that there are uninterrupted pathways for both electrons and holes to be transported to the electrodes. Despite the beneficial effect of an increased amount of donor on the optical properties, in view of the electrical properties, the amount of donor material cannot be increased continuously. The active layer ingredients should have a homogeneous structure to ensure an adequate transport of the carriers under the energy levels. Chemically, Raman spectroscopy results showed a domination of molecular dynamics by vibrations originating from P3HT, both over the PCBM acceptor and additional donors (dyes).

**Figure 13:**
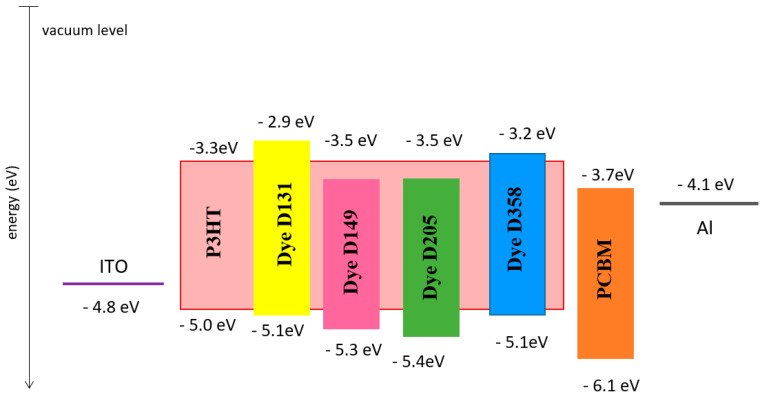
Energy diagram for the investigated materials in the context of the use of their ternary organic cells.

Based on the HOMO levels obtained from the UPS spectra and the energy gap obtained from the absorption spectrum, we can determine the energy diagram for potential ternary solar cells (Figure 13). The HOMO level was determined by determination from Figure 11b (comparison of the cut-offs for all samples) Fermi level position and Fermi level position relative to the HOMO level (VB). These quantities were subtracted from the He-I radiation energy radiation of 21.2 eV [51,52]. It should be further considered here that the number of P3HT molecules decreases, which molecule receives the holes of the excited molecules quenching the possible photoluminescence from the dyes. From the data shown in Figure 13, it is further evident that the acceptor molecules receive excited electrons via photoluminescence.

Due to the similar energy levels of both donors, it can be concluded that the transport of charge carriers will be supported by an additional donor. The application of the materials and the fabrication of the cells require further specified research.

## 5. Summary

The optical properties, morphology, and molecular states of the selected dyes as well as their ternary mixtures with a donor (P3HT) and an acceptor (PCBM) have been studied in this publication. The aim was to determine their potential for use in the active layer of ternary organic cells. Considering the absorption spectra, all the considered dyes are excellent materials for active layer enrichment, as they have a wide absorption spectra in the region of maximum solar radiation. All dyes also exhibit luminescence, so they are able to produce a stable excited state. Analysis of thin film morphology indicates that D205 dye is not necessarily suitable for the application due to the lack of homogeneity of the thin film at nanometer level. The other dyes with donor (P3HT) and acceptor (PCBM) form very consistent layers and in terms of surface morphology are most optimistic for three components active layer. The resulting energy levels structure also places itself in the range supporting charge transport. The presented properties of the considered compounds indicate the high applicability for implementation in organic ternary solar cells. Tests have been conducted in air but research in vacuum is planned as polymer photovoltaics are likely to be used in space.

## Figures and Tables

**Table 1:** Refractive indices and extinction coefficients values for wavelength λ=633 nm investigated materials.

Compound	Refractive Index for 633 nm	Extinction Coefficient for 633 nm
D131	1.81	0.171
D149	2.04	0.112
D205	2.03	0.101
D358	1.94	0.0385

**Table 2:** Roughnes Rq parameter values for the investigated materials.

Layer Composition	Roughness Rq Parameter (nm)
P3HT:PCBM 1:1	0.640
D131:P3HT:PCBM	0.379
D149:P3HT:PCBM	0.323
D205:P3HT:PCBM	0.528
D358:P3HT:PCBM	0.248

## Data Availability

The data presented in this study are available on request from the corresponding author.

## Supplementary Materials

The following are available online at https://www.mdpi.com/article/10.3390/polym13234099/s1, Figure S1: AFM profiles for the tested mixtures (a) P3HT:PCBM (b) dye D131:P3HT:PCBM (c) dye D149:P3HT:PCBM (d) dye D205:P3HT:PCBM (e) dye D358:P3HT:PCBM, Table S1: Information about materials, Table S2: Thicknesses of investigated layers determined by spectroscopic ellipsometry.
