# Supplementary material for: Investigation of Dye Dopant Influence on Electrooptical and Morphology Properties of Polymeric Acceptor Matrix Dedicated for Ternary Organic Solar Cells
Source: Polymers (Basel). 2021 Nov 25;13(23):4099. doi: 10.3390/polym13234099 (PMC8658881; doi:10.3390/polym13234099)
Supplement: Supplementary file 1 [file polymers-13-04099-s001.zip › polymers-1449805-supplementary.pdf]

Supplementary Materials

Investigation of Dye Dopant Influence on Electrooptical and Morphology Properties of Polymeric Acceptor Matrix Dedicated for Ternary Organic Solar Cells

Gabriela Lewińska [1,*], Piotr Jeleń [2], Jarosław Kanak [1], Łukasz Walczak [3], Robert Socha [4], Maciej Sitarz [2], Jerzy Sanetra [†] and Konstanty W. Marszałek [1]

1 AGH University of Science and Technology, Faculty of Computer Science, Electronics and Telecommunication, 30 Mickiewicza Ave., 30-059 Krakow, Poland; kanak@agh.edu.pl (J.K.); marszale@agh.edu.pl (K.W.M.)
2 Department of Silicate Chemistry and Macromolecular Compounds, Faculty of Materials Science and Ceramics, AGH University of Science and Technology; 30-059 Krakow, Poland; pjelen@agh.edu.pl (P.J.), msitarz@agh.edu.pl (M.S.)
3 Science & Research Division, PREVAC sp. z o.o., Raciborska 61, 44-362 Rogow, Poland; lukasz.walczak@prevac.pl
4 Jerzy Haber Institute of Catalysis and Surface Chemistry, Polish Academy of Sciences, Niezapominajek 8, 30239 Krakow, Poland; robert.socha@ikifp.edu.pl
* Correspondence: glewinska@agh.edu.pl; Tel.: + 48-692-376-639
† The author Jerzy Sanetra is retired ; jsanetra@agh.edu.pl

The table presents data on the tested materials.

Table S1. Information about materials (*based on manufacturer's data [1])

Abbreviation	Full chemical name	Empirical Formula	Molecular weight (u)	Purity* HPLC (%)
Dye D131	2-cyano-3-[4-[4-(2,2-diphenylethenyl)phenyl]-1,2,3,3a,4,8b-hexahydrocyclopent[b]indol-7-yl]-2-propenoic acid	$C_{35}H_{28}N_2O_2$	508.61	>95
Dye D149	5-[[4-[4-(2,2-diphenylethenyl)phenyl]-1,2,3-3a,4,8b-hexahydrocyclopent[b]indol-7-yl]methylene]-2-(3-ethyl-4-oxo-2-thioxo-5-thiazolidinylidene)-4-oxo-3-thiazolidineacetic acid	$C_{42}H_{35}N3O_4S_3$	741.94	98
Dye D205	5-[[4-[4-(2,2-diphenylethenyl)phenyl]-1,2,3,3a,4,8b-hexahydrocyclopent[b]indol-7-yl]methylene]-2-(3-octyl-4-oxo-2-thioxo-5-thiazolidinylidene)-4-oxo-3-thiazolidineacetic acid	$C_{48}H_{47}N_3O_4S_3$	826.10	97
Dye D358	5-[3-(carboxymethyl)-5-[[4-[4-(2,2-diphenylethenyl)phenyl]-1,2,3,3a,4,8b-hexahydrocyclopent[b]indol-7-yl]methylene]-4-oxo-2-thiazoli dinylidene]-4-oxo-2-thioxo-3-thiazolidinedodecanoic acid	$C_{52}H_{53}N_3O_6S_3$	912.19	95

Dispersion relationships are discussed below [2].

Tauc - Lorentz absorption formula is as follows Lorentz [3,4]

$$\varepsilon_2 \propto \left(E - E_g\right)^2 / E_g^2$$

and Cody – Lorentz model is shown by the equation [5]

$$\varepsilon_2 \propto \left(E - E_g\right)^2$$

Detalied Cody absorption formula is describe by

$$\varepsilon_{n\_CL} = \varepsilon_{n1} + i\varepsilon_{n2}$$

where

$$\varepsilon_2(E) = \begin{cases} \dfrac{E_1}{E} \exp\left(\dfrac{E - E_{gn} - E_{tn}}{Eun}\right) & 0 < E \leq E_{gn} + E_{tn} \\ G(E)L(E) = \dfrac{\left(E - E_{gn}\right)^2}{\left(E - E_{gn}\right)^2 - E_{pn}{}^2} - \dfrac{A_n E_{on} \Gamma_n E}{(E - E_{on})^2 + \Gamma_n^2 E^2} & ; E > E_{gn} - E_{tn} \end{cases}$$

$E_1 = (E_{gn} + E_n)\mathrm{G}(E_{gn} + E_{tn})\mathrm{L}(E_{gn} + E_{tn})$

$$\varepsilon_{n1} = \frac{2}{\pi} P \int_0^\infty \frac{\xi \varepsilon_{n2}(\xi)}{\xi^2 - E^2} d\xi$$

Where

n≡oscilator , E_g ≡ bandgap, $\varepsilon_1, \varepsilon_2$ ≡ the real and imaginary parts of the dielectric function, G(E) ≡nearbandgapfunction, L(E) ≡ Lorentz oscillator function, E_t, E_p transition energies

The detailed Tauc-Lorentz absorption formula is desribed by

$$\varepsilon_2(E) = \frac{A E_0 \Gamma \, (E - E_g)^2}{E[(E^2 - E_0^2)^2 - \Gamma^2 E^2]} \Theta \, (E - E_g)$$

E_g is the band gap of the material, A is a refactor, which includes the optical transition matrix elements., a Θ is the Heaviside Theta function, E_0 is the peak in the joint density of states, Γ is he broadening parameter.

The real part of the TL dielectric function is

$$\varepsilon_1(E) = n^2(E) - k^2(E)$$

Table S2. Thicknesses of investigated layers determined by spectroscopic ellipsometry

	Single dye thicknesses (nm)	Dye: P3HT: PCBM (nm)
Dye D131	108.0	49.1
Dye D149	82.0	48.4
Dye D205	80.5	47.9
Dye D358	65.9	50.4

In the Figure S1, the profiles obtained from AFM measurements for the studied mixtures are shown.

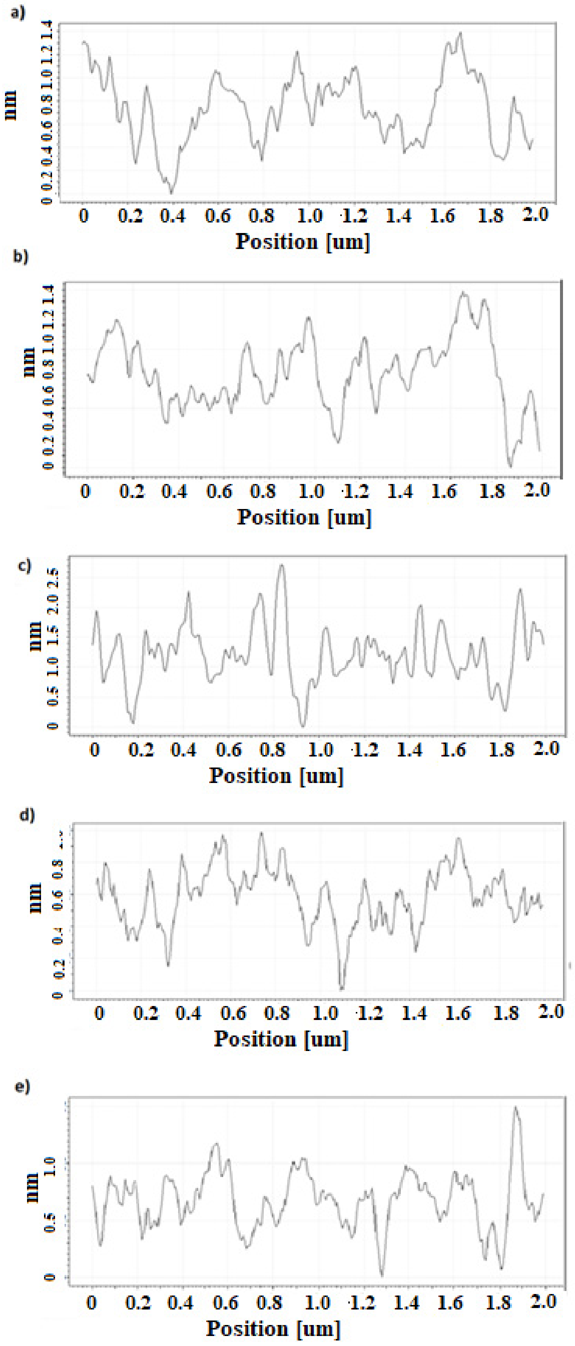

Figure S1. AFM profiles for the tested mixtures a) P3HT:PCBM b) dye D131:P3HT:PCBM c) dye D149:P3HT:PCBM d) dye D205:P3HT:PCBM e) dye D358:P3HT:PCBM

References

1. Merck | Nauki biomedyczne | Substancje chemiczne dla przemysłu i laboratorium | eSklep Available online: https://www.merckmillipore.com/PL/pl (accessed on Nov 9, 2021).

2. Woollam Co, J. Complete EASE, Software Manual. **2007**.

3. Jellison, G.E.; Modine, F.A. Parameterization of the optical functions of amorphous materials in the interband region. *Appl. Phys. Lett.* **1996**, *69*, 371–373.

4. Jellison, G.E.; Jr.; Merkulov, V.I.; Puretzky, A.A.; Geohegan, D.B.; Eresa, G.; Lowndes, D.H.; Caughman, J.B. Characterization of thin-film amorphous semiconductors using spectroscopic ellipsometry. *Thin Solid Films* **2000**, *377–378*, 68.

5. Ferlauto, A.S.; Ferreira, G.M.; Pearce, J.M.; Wronski, C.R.; Collins, R.W.; Deng, X.; Ganguly, G. Analytical model for the optical functions of amorphous semiconductors from the near-infrared to ultraviolet: Applications in thin film photovoltaics. *J. Appl. Phys.* **2002**, *92*, 2424–2436.
